# Is dependence on activities of daily living an indicator of social isolation among old people living alone in public housing?

**DOI:** 10.1192/j.eurpsy.2025.1741

**Published:** 2025-08-26

**Authors:** S. P. V. Martins, J. Guedes, H. Alves, M. Videira, I. Machado, S. Melo, F. Melo

**Affiliations:** 1 ISSSP, Porto Institute of Social Work; 2 CESPU, CRL - Cooperativa de Ensino Superior, Politécnico e Universitário - iHealth4Well-being Research Unit; 3 CINTESIS@RISE, Faculty of Medicine, University of Porto; 4 CLISSIS-Centro Lusíada de Investigação em Serviço Social e Intervenção Social; 5 LIAAD-INESC TEC; 6 Institute of Sociology, University of Porto; 7 Domus Social Company-Porto City Council, Porto, Portugal

## Abstract

**Introduction:**

Social isolation (SI) is defined as the lack of social contact or support. Older adults have a higher risk of social isolation because of the changes in health and social relationships that can occur during ageing. Several studies have shown that SI is independently associated with poorer physical and mental health and worsened quality of life. However, limited evidence is available on SI predictors in old public housing populations.

**Objectives:**

To assess the risk of SI and dependency in Basic and Instrumental Activities of Daily Living (BADL; IADL) in a sample of older people living alone in public housing. To identify predictors of SI, namely whether ADL dependency is one of them.

**Methods:**

As part of the ongoing “Porto Importa-se” project, this study included a sub-sample of older persons aged 70 years and over living alone in public housing communities in Porto City, Portugal. All participants were assessed with a comprehensive multidimensional assessment protocol, which encompassed the Barthel and Lawton Indexes (BADLs and IADLs dependency) and the Lubben Social Network Scale-6 (SI risk). Loneliness was measured with a categorical question. A multiple logistic regression model was performed to identify predictive factors for SI. Odds Ratio (OR) and its 95% Confidence Interval (95%CI) were calculated. A p<0.05 was considered statistically significant.

**Results:**

The final sample (n=716) was namely female (84%), with an average age of 80.4 years (SD=6.2). Around 36% presented a risk of SI, and 24% reported feeling lonely almost always to always. About 53% had moderate dependency on IADLs, and 11% dependency on BADLs. The proportion of participants dependent on BADLs and at risk of SI is more than double the proportion of cases considered not to be at risk (17%v.s.8%; p<0.001). Similarly, the proportion of cases considered to be severely dependent on IADLs and at risk of SI is about four times higher than the proportion of cases considered not to be at risk (13%v.s.3%; p<0.001). Based on the logistic regression model, severe dependence on IADLs (OR=5.16, 95%CI[2.37;11.24], p<0.001) and loneliness (OR=2.87, 95%CI[2.02;4.09], p<0.001) were significant predictors of the risk of SI. The model has a modest explanatory power (Nagelkerke R^2^=0.126).

**Conclusions:**

The rate of SI found in this study aligns with the results reported in other studies with similar objectives. The identification of loneliness and dependence in ADL as predictors of SI also complies with previous studies. These results reinforce the importance of monitoring elderly people who find themselves alone and dependent on the fulfilment of their ADLs more closely.

This work was supported by National Funds through FCT - Fundação para a Ciência e a Tecnologia,I.P., within CINTESIS, R&D Unit (reference UIDP/4255/2020)

**Disclosure of Interest:**

None Declared

